# High Levels of PD-L1^+^ and Hyal2^+^ Myeloid-derived Suppressor Cells in Renal Cell Carcinoma

**DOI:** 10.15586/jkcvhl.v9i2.208

**Published:** 2022-04-16

**Authors:** Sergei Kusmartsev, Elizabeth Kwenda, Paul R. Dominguez-Gutierrez, Paul L. Crispen, Padraic O’Malley

**Affiliations:** Department of Urology, University of Florida, Gainesville, FL, USA

**Keywords:** renal cell carcinoma, cancer immune microenvironment, myeloid-derived suppressor cells, Hyal2

## Abstract

Renal cell carcinoma (RCC) patients frequently have increased number of immunosuppressive myeloid cells in circulation. High number of myeloid-derived suppressor cells (MDSCs) in the blood are associated with immune suppression as well as with cancer-related inflammation which drives the mobilization of myeloid cells to tumor tissue. Here, we show that peripheral blood from a previously untreated RCC patient has increased the number of monocytic CD33^+^CD11b^+^ MDSCs, which also co-expressed PD-L1 and membrane-bound enzyme hyaluronidase 2 (Hyal2). PD-L1 expression is associated with immune suppression, whereas expression of Hyal2 is associated with inflammation, because Hyal2^+^ myeloid cells can degrade the extracellular hyaluronan (HA), leading to the accumulation of pro-inflammatory HA fragments with low molecular weight. These findings implicate the potential involvement of monocytic MDSCs in both tumor-associated immune suppression and cancer-related inflammation. Analysis of organotypic tumor-tissue slice cultures prepared from cancer tissue of the same patient revealed the significant presence of PD-L1^+^ HLA-DR^+^ macrophage-like or dendritic cell-like antigen-presenting cells in tumor stroma. Interestingly, stroma-associated PD-L1^+^ cells frequently have intracellular hyaluronan. Collectively, data presented in this study suggest that the interplay between tumor-recruited myeloid cells and stromal HA may contribute to the inflammation and immune tolerance in kidney cancer.

## Introduction

Immune checkpoint inhibitors have improved the treatment of a broad spectrum of cancers including renal cell carcinoma (RCC), metastatic melanoma, and non-small lung cancer. These humanized monoclonal antibodies target inhibitory receptors (e.g., CTLA-4, PD-1, LAG-3, TIM-3) and ligands (PD-L1) expressed on T lymphocytes, antigen-presenting cells, and tumor cells and elicit an anti-tumor response by stimulating the immune system (1, 2). However, both cancer-related inflammation and tumor-associated immune suppression frequently override the anti-tumor immune response (3, 4). Cancer patients, including patients with RCC, frequently have increased the number of immunosuppressive myeloid cells such as myeloid-derived suppressor cells (MDSCs) (5, 6). Thus, MDSCs isolated from the blood of patients, but not from healthy donors, were capable of suppressing antigen-specific T-cell responses *in vitro* through the secretion of reactive oxygen species and nitric oxide upon interaction with cytolitic T-lymphocytes (CTL). More recently, it was demonstrated that monocytic MDSCs in patients with bladder cancer, in contrast to healthy donors, frequently co-express membrane-bound enzyme hyaluronidase 2 (Hyal2). Here, we show an increased presence of PD-L1^+^ myeloid cells in both peripheral blood and tumor tissue from a previously untreated patient with clear cell RCC. Furthermore, tumor-infiltrating PD-L1^+^ myeloid cells express a marker of antigen-presenting cells HLA-DR and show a significant amount of internalized hyaluronan, indicating the possible contribution of stroma and tumor-associated hyaluronan (HA) in the modulation of immune function of myeloid cells including antigen-presentation.

## Case Report

The 67-year-old man diagnosed with non-metastatic cell clear RCC with no prior cancer treatment history underwent radical nephrectomy. Clinical samples including peripheral blood and freshly excised kidney tumor tissue were transferred to the research laboratory for analysis. Clinical samples were collected from the patient after obtaining written informed consent. All samples were obtained according to federal guidelines and as approved by the University of Florida institutional review board (IRB). Final pathology demonstrated pT3a N0 clear cell RCC with ISUP grade 3, negative surgical margins, and no presence of adverse markers such as sarcomatoid, rhabdoid, or necrosis.

Peripheral blood mononuclear cells (PBMCs) were isolated by gradient density centrifugation using Lymphoprep (Accu-Prep, 1.077 g/mL, Oslo, Norway). CD11b myeloid cells were purified from PBMCs by positive selection using the anti-CD11b microbeads and columns (Miltenyi Biotec, Bergisch Gladbach, North Rhine-Westphalia, Germany.). Immunofluorescent staining and analysis were performed according to the previously described protocol (7, 8).

**Figure 1:**
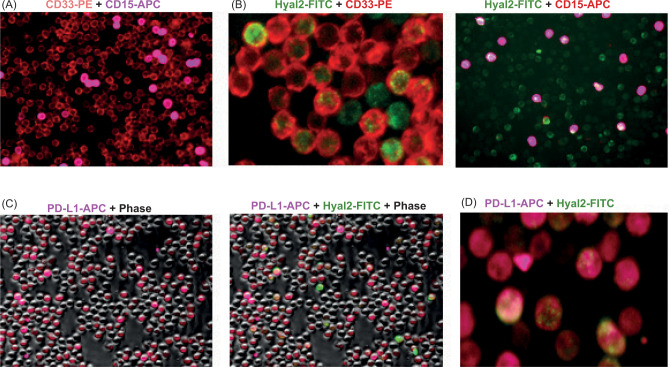
The increased presence of PD-L1 and Hyal2-expressing myeloid cell subsets in the patient’s peripheral blood. CD11b myeloid cells were isolated from the peripheral blood using magnetic beads. Freshly isolated cells were stained with CD33-PE, CD15-PE, and Hyal2-FITC antibodies (images A, B), or with PD-L1-APC and Hyal2-FITC antibodies (images C, D). Representative IF images are shown.

To examine whether myeloid cells in RCC express the membrane-bound enzyme hyaluronidase 2 (Hyal2), we have isolated CD11b^+^ myeloid cells from the peripheral blood of a previously untreated patient with RCC. First, we looked at the presence of CD33^+^ monocytic and CD15^+^ granulocytic MDSCs. Data presented in Figure 1A demonstrate the high level of CD33 expression and relatively low expression of CD15 among blood-derived myeloid cells. Similar to human bladder cancer, CD33^+^ in a patient with RCC, also co-expressed membrane-bound enzyme Hyal2 (Figure 1B, left image). Furthermore, Hyal2^+^ myeloid cells have also co-expressed the immunosuppressive ligand PD-L1 (Figure 1C and D).

In addition to peripheral blood, the Hyal2-expressing myeloid cells can also be found in tumor tissue (8). To examine the tumor-associated myeloid cells in RCC tissue, we prepared the organotypic cancer tissue slices using freshly excised tumor tissue from the same patient. The organotypic precision-cut tissue slices, 2–4 mm in diameter and 300-micron thick, were produced using a Compresstome Vibratome VF-300-0Z (Natick, MA, USA). After cutting, tissue slices were placed into 24-well cell culture plates in complete RPMI-1640 medium supplemented with 10% FBS and antibiotics and cultured at 37° C in a humidified CO_2_ incubator. The organotypic tumor tissue slice technique, which has been significantly used during recent years, provides a novel approach for studying the stroma-immune interactions and may offer a novel avenue to reduce the translational gap. Thus, cultures of precision-cut tissue slices prepared from freshly excised tissue, create nearly ideal conditions to explore the interaction between tumor stroma and immune cells. Once tumor slices are placed in a culture flask or plate, they start the formation of an adherent stroma which includes an extracellular matrix with attached fibroblasts, macrophages, and other immune cells.

**Figure 2:**
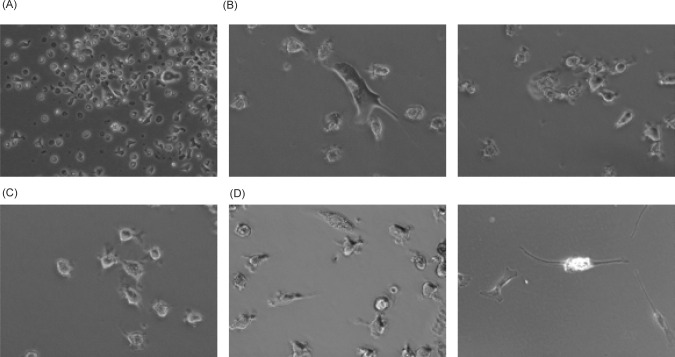
Tumor-infiltrating immune cells interact with cancer-associated fibroblast-like cells. Live imaging (before fixation). Representative bright-field images of tumor stroma from the same patient are shown (images A–D).

**Figure 3:**
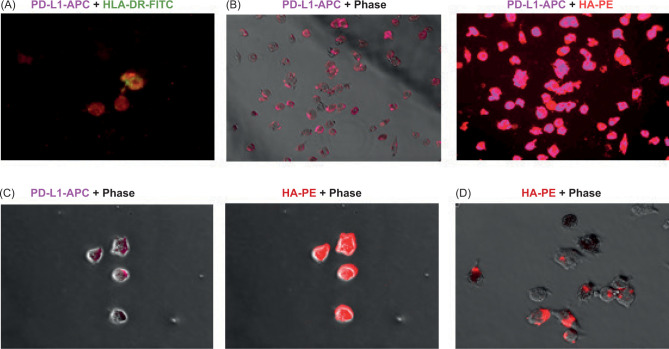
Visualization of intracellular HA in tumor-infiltrating PD-L1^+^ myeloid cells. The human cancer tissue slices were cultured for 7 days. Non-adherent cells were carefully removed from the plate. Plate with remaining adherent cells washed with PBS and fixed with 4% formaldehyde. Plate-bound cells were stained with HLA-DR-FITC (image A) and PD-L1-APC (images A, B, C) antibodies. To visualize the tumor-produced HA, biotinylated HA-binding protein and PE-labeled Streptavidin were subsequently added (images B, C, D). Representative images are shown.

Live imaging of stroma in RCC tissue slice cultures before fixation showed a significant presence of both irregularly shaped fibroblast-like large cells, and smaller round shaped macrophage-like or dendritic cell-like cells (Figure 2A–D). Moreover, the smaller cells were observed in the close proximity of fibroblast-like cells (Figure 2B), suggesting the potential interaction between those cells. After fixation and washing of tumor tissue slices with PBS, we stained the remaining adherent stromal cells for the PD-L1. Data presented in Figure 3A and B demonstrate that majority of macrophage-like or dendritic-cell-like cells in RCC stroma express PD-L1. Co-expression of HLA-DR by these cells (Figure 3A) supports the idea that these PD-L1^+^ cells belong to the antigen-presenting cells. Also, staining of RCC stroma for HA revealed the PD-L1^+^ cells have a marked presence of intracellular HA (Figure 3B–D).

**Figure 4:**
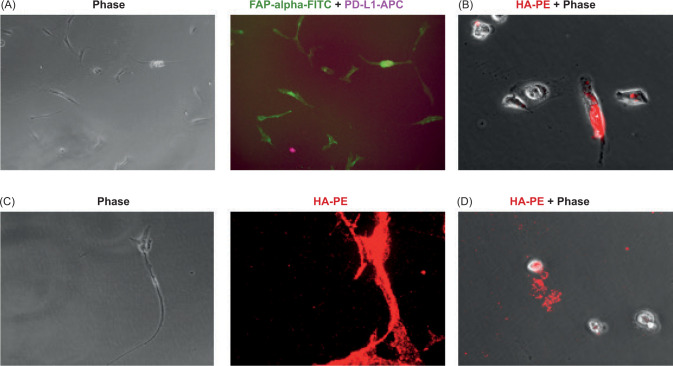
Detection of cancer-associated fibroblasts in kidney tumor stroma. The tissue slices were cultured for 7 days. Non-adherent cells were carefully removed from the plate. Plate with remaining adherent cells washed with PBS and fixed with 4% formaldehyde. Plate-bound cells were stained with FAP-alpha-FITC and PD-L1-APC (image A) antibodies. To visualize the tumor-produced HA, biotinylated HA-binding protein and PE-labeled Streptavidin were subsequently added (images B, C, D). Representative images are shown.

Additional analysis revealed that stromal fibroblast-like cells expressed fibroblast-specific marker FAP-alpha (Figure 4A). These data are consistent with previous reports demonstrating the presence of FAP-alpha cancer-associated fibroblasts in RCC tissues (9, 10). Staining of stroma for the HA showed (Figure 4B–C) that localization of cancer-associated fibroblasts (CAFs) in RCC stroma is associated with HA (red) suggesting that CAFs contribute to the HA in the RCC tumor microenvironment.

Patient remained clear of recurrent disease as of last follow up in December 2021, approximately 18 months post-op.

## Discussion

The enhanced HA metabolism has previously been described in several major subtypes of RCC such as clear cell, papillary, and chromophobe renal carcinomas (11). Thus, the median transcript levels of hyaluronic acid synthase 1 (HAS1) and major HA receptors CD44 and RHAMM were elevated 3 to 25-fold in those tumor tissues when compared with normal tissues. Both synthesis and degradation of HA in various types of cancer are frequently heightened (12–14). Strong HA degradation in tumor tissues can be partially explained by increased mobilization of Hyal2^+^ myeloid cells in cancer patients following elevated number of Hyal2^+^ myeloid cells being detected in the peripheral blood (8). Upon recruitment to the tumor, Hyal2^+^ expressing myeloid cells capable of degrading extracellular HA into small fragments, promoting the accumulation of HA fragments with low molecular weight (LMW) (20 kDa). Accumulation of LMW-HA fragments in tumor tissue has been associated with enhanced production of multiple inflammatory and pro-angiogenic factors. Since tumor stroma is rich in HA, it is plausible that tumor stroma is involved in the regulation of an anti-tumor immune response. However, the exact mechanisms of stroma-immune interactions in cancers including RCC remain largely unknown. Our data demonstrate the frequent co-localization of HA and fibroblast-like cells in RCC stroma.

Detectable cellular HA in the RCC tumor microenvironment was associated with higher tumor grades in patients and has been identified as a prognostically unfavorable subgroup among low-grade carcinomas (15). Here, we show that peripheral blood of previously untreated RCC patient is enriched for the monocytic CD33^+^ MDSCs. These myeloid cells co-express immunosuppressive ligand PD-L1 as well as membrane-bound enzyme Hyal2. High levels of PD-L1 are associated with immune suppression (16–19), whereas Hyal2 expression indicates the involvement of these cells in the process of HA degradation which contributes to cancer-related inflammation (8, 20–22). Upon recruitment to the tumor tissue, tumor-associated MDSCs continuously differentiate into immunosuppressive antigen-presenting cells such as macrophages (8, 23). Multiple phenotypes of RCC tumor-associated macrophages (TAMs) have also been reported (23). Functionally, TAMs play diverse roles in tumor growth by mediating immunosuppression, promoting tumor angiogenesis, inducing tumor migration and metastasis, and enhancing resistance to chemotherapy and radiotherapy. Data presented in this article indicate that macrophage-like HLA-DR^+^ cells in RCC stroma frequently express immunosuppressive PD-L1 ligand and also show significant amounts of internalized tumor-associated HA. Both tumor epithelial cells and CAFs were recently identified as the main sources of HA in the tumor microenvironment (24). Taken together, this work demonstrates that stromal HA metabolism in the RCC tumor microenvironment may contribute to the regulation of anti-tumor immune response through modulation of tumor-infiltrating antigen-presenting cells (APCs).
